# Is the Mosquito a Factor in Disseminating Yellow Fever?

**Published:** 1902-07

**Authors:** E. G. Ferguson

**Affiliations:** Macon, Ga.


### IS THE MOSQUITO A FACTOR IN DISSEMINATING YELLOW FEVER? *

BY E. G. FERGUSON, M.D.,

MACON, GA.

Numerous experiments have recently been indulged in to prove that the mosquito conveys the contagion of yellow fever. After having indulged its appetite on a case, it inoculates those whom it afterwards bites. Nothing could be more preposterous as a proposition to offer an intelligent medical world, and to prove its absurdity I shall give the history of two epidemics in which I served, one in 1876 in Brunswick, on the coast of Georgia, and the other in 1878 in Vicksburg, in the Mississippi Valley. In the one in Brunswick in 1876 I served my apprenticeship. Two vessels from Havana with sick on board, in ballast with mud or earth taken in the neighborhood of Havana, discharged their cargoes at the docks in Brunswick. The sick had their clothes washed by washerwomen who were on the lookout for vessels coming to port to get sailors' washing, some living along the docks, while others lived on the outskirts of the town. These were the first to contract the disease; from this source of infection it spread to all parts of the town. All are familiar with the fact that when a nigger is sick all his friends will crowd the house, and through fomites convey infectious diseases for miles. In that epidemic I noticed for

* Read before the Macon Medical Society, May 20, 1902.

over a month or more before I was stricken down with the disease, that, when I would defecate in the woods, and I nearly always did so, the characteristic odor of the disease would be quite manifest to my nostrils. So patent was this that others observed it as well. The mayor of Brunswick, Tom Davenport, told me he had detected the odor for quite a time before he was taken down with the disease. The system becomes saturated with the infection for a month or more before the fever develops, and if a mosquito bites him it was the insect conveyed the disease. In Brunswick hundreds nursed their own sick during their period of illness, and then went to the relief of their friends, spending many days and nights at their bedsides, and never took the disease. Why did not the mosquito bite convey the disease to all alike? She, the female mosquito, must have been roaming around over the sick as well as the well.

The Rev. John Simmons will, I think, bear me out on my assertions regarding the origin of the disease and the number of cases that escaped. There is one thing that is doubtless quite patent to his mind, that, where we boarded at Charlie Moor's, we had to live on corn bread, sweet potatoes, no salt, and water for a week. The parson said he did not object to the diet, but entered his protest because there was no salt.

On the 12th of July, 1878, the John D. Porter, a tug boat, left New Orleans, having a tow of several barges with their respective crews aboard, many of whom had been exposed to the fever then under good headway in that city. The tug landed at Baton Rouge, on the Mississippi, and put off one of the hands who was sick with yellow fever, presumably not knowing the nature of the illness the hand was laboring under. Going on to Natchez, another was put off, and at Vicksburg another. The tug went on to Memphis, where they were refused landing by the quarantine officer. Each one of the places became infected with the disease, and from each of these centers it spread into the surrounding country, following the avenues of commerce, until the whole Mississippi Valley was a hotbed of the disease. Could it be possible that the tug boat, John D. Porter, running at the rate of twenty miles a day, carried a load of infected mosquitoes and infected the whole valley with the disease, when a breeze was constantly blowing over the tug and barges fore and aft, as well as being exposed to winds from all points of the compass during the voyage? Would it be likely that these infected mosquitoes could retain a hold on the tug and barges and not be blown away under such adverse circumstances, but at the proper time light off at these respective places that I before mentioned and infect the community where they landed? Some time that season, when the fever was gaining headway, those experienced in the treatment of the disease were loudly called for. I responded to a telegram and went to Vicksburg. Arriving in Vicksburg on Saturday night, too late to be assigned to duty by the Howard Association, I repaired to the Washington Hotel and slept in a bed in which a man had died of the fever, with no change of sheets or washing black vomit from the wall. Sunday morning I reported for duty to the Howard Association, and was furnished with a pair of horses and landau, having instructions to go where I pleased; I would find plenty to do. At 11 o'clock that night I had 144 cases. For two weeks I slept in the carriage, being waked up at night by my driver at the respective houses where my patients were. Being a visiting physician, I was requested by the President of the Howard Association to furnish a list of my patients in the city to the secretary of the Howards, and collect nurses and supplies and proceed to Bovina, as they were crying out for help. Stayed in Bovina one week, when the local physicians were able to attend to the cases, and returned to the city. Was sent to Smith's station, a few miles beyond Bovina, where I remained a few days and returned to the city and reported for further duty. Dr. Harris, of Galveston, Texas, stationed at Bolton, wired for help, as he had 600 cases, the local physicians sick, and was unable to keep up with the work. Assembled together thirty nurses and took seven tons of ice, with other supplies, and proceeded to Bolton on the cars. We had to pass through Edwards to get to Bolton. Before entering Edwards we were confronted with a large placard, on which was written, "Stop not, under penalty of death!" A cannon pointing in our direction, with a man at the lanyard and soldiers were on watch. A cordon of soldiers surrounded the village. This was shotgun quarantine. The train did not stop; we went on to Bolton. There was not a case of fever in Edwards. The mosquito smelt the powder. After two weeks in Bolton I returned to Vicksburg, and reported to the Howards that the resident physicians were up and able to work. A call from McNairs, which was eighteen miles in the country from Bolton, was assigned to me. Twenty nurses and supplies had to be taken to McNairs. Teams were furnished at Bolton, and on my arrival there, after again passing through Edwards, I transferred my nurses and supplies to the wagons. We proceeded to Raymond, which is half way between Bolton and McNairs, and found a rope stretched across the road leading into Raymond, with soldiers guarding it, a cordon surrounding the village. We were ordered not to approach any nearer than fifty yards. Had the board of health assembled and persuaded them that there was no danger if we would go through on a trot. The rope was removed and we proceeded on our way to McNairs, which was nine miles distant. There was not a case in Raymond. Shotgun quarantine kept the mosquitoes out. Nine miles from Raymond was McNairs, where there were ninety-six cases. To sum the matter up, the John D. Porter left mosquitoes at Baton Rouge, at Natchez and Vicksburg, but the shotgun quarantine kept them out of Edwards and Raymond. In my estimation the mosquito is as big a piece of humbuggery as ever was palmed off on a nation. As soon as the system becomes saturated with the infection, if a non-immune or an immune, the person goes down with the disease.

